# Reduction in Measurement Error: Barraza-Villarreal et al. Respond

**DOI:** 10.1289/ehp.11804R

**Published:** 2008-10

**Authors:** Albino Barraza-Villarreal, Consuelo Escamilla-Nuñez, Leticia Hernández-Cadena, Isabelle Romieu, Silvia Ruiz-Velasco, Jordi Sunyer

**Affiliations:** Instituto Nacional de Salud Publica de México, Cuernavaca, Morelos, México, E-mail: abarraza@insp.mx; Instituto de Investigaciones en Matemáticas Aplicadas y en Sistemas, Universidad Nacional Autónoma de México, México, D.F., México; Environmental Epidemiological Research Centre, Institut Municipal d’Investigació Médica, Barcelona, Spain

We thank Barnett for his comments on our article (Barraza-Villarreal et al. 2008), in which we reported associations between ambient air pollution and adverse lung function outcomes in a cohort of schoolchildren in Mexico City, Mexico. In the last several years, the adverse effects of air pollution on lung function, such as decrement in forced expiratory volume in 1 sec (FEV_1_) has been clearly demonstrated (Gauderman et al. 2007; Romieu et al. 1997). Before our study, there were reports of associations between cumulative particulate matter [PM < 10 μm (PM_10_) and < 2.5 μm (PM_2.5_ ) in aerodynamic diameter] and gaseous (ozone, sulfur dioxide, and nitrogen dioxide) air pollutant exposure and decrease in lung function in other studies (Downs et al. 2007; Romieu et al. 2006). Replication of these findings in different populations under different conditions of exposure is an important aspect of epidemiologic research, with consistency of results strengthening the weight of evidence for a true association between exposure and outcome.

However, air pollution exposure assessment is always a critical factor in environmental epidemiology. Like other studies of air pollution and lung health, our study (Barraza-Villarreal et al. 2008) relied on ecologic rather than personal indicators of exposure. Exposure misclassification due to the use of fixed-site ambient monitors rather than personal dosimeters is likely to underestimate rather than overestimate the effect of air pollution on lung function.

In his letter Barnett mentions that “the apparent stronger association between reduced FEV_1_ and cumulative exposure over 1–5 days may be due in part to a reduction in measurement error of particulate matter < 2.5 μm (PM_2.5_) and not a true cumulative effect.” He attempted to verify this assertion by carrying out a simulation study; however, we see several problems with it. First, in his simulations, Barnett assumed a normal distribution (Figure 1). Several distributions have been reported as adequate for PM_2.5_, among them log-logistic, log-normal, and gamma. Using the data from our study (Barraza-Villarreal et al. 2008), we carried out an exercise similar to Barnett’s, but we fitted different distributions (data not shown). The one that best fit our data was the gamma distribution. Second, when considering cumulative exposure, it is important to take into account the correlation between the observations on consecutive days; it is not enough to simulate from a distribution and then add the exposure. The models presented by Barnett did not take into account this correlation. Third, we reproduced the simulation of FEV_1_ as presented by Barnett (data not shown) and observed that it could produce negative value for FEV_1_ because it does not take into account the correlation of observations within children, although a sample size for each child was simulated and an artificial mixed model was fitted.

In conclusion, because Barnett’s simulation of PM_2.5_ was based on a normal distribution, it does not reproduce the original structure of our data (Figure 1) (Barraza-Villarreal et al. 2008); therefore, the conclusions obtained are not applicable.

## Figures and Tables

**Figure 1:**
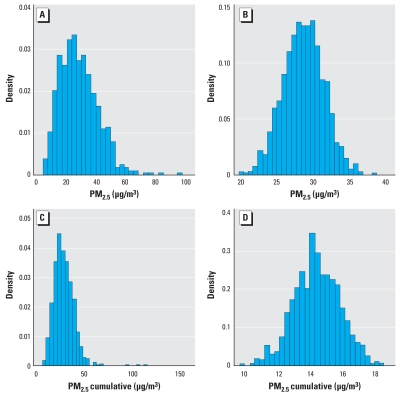
Same day (*A,B*) and 2-day cumulative (*C,D*) PM_2.5_ distributions. (*A,C*) original data. (*B,D*) Simulated data.
